# An Unusual Case of Paraganglioma of the Broad Ligament Presenting as Cystic Mass

**DOI:** 10.1155/2016/1041989

**Published:** 2016-10-13

**Authors:** Siddhi Gaurish Sinai Khandeparkar, Maithili Mandar Kulkarni, Vandana Gaopande, Avinash Joshi, Pushpalata Naphade

**Affiliations:** ^1^Department of Pathology, Shrimati Kashibai Navale Medical College and General Hospital, Pune, India; ^2^Department of Obstetrics and Gynaecology, Shrimati Kashibai Navale Medical College and General Hospital, Pune, India

## Abstract

In clinical practice, broad ligament (BL) tumors are seldom encountered. Paraganglioma of the BL is exceedingly rare entity. Here we present an unusual case of broad ligament paraganglioma, presenting as a cystic mass, in a 50-year-old postmenopausal female. A high degree of suspicion along with detailed immunohistopathological work-up is needed for arriving at an accurate diagnosis.

## 1. Introduction

In clinical practice, broad ligament (BL) tumors are seldom encountered [1]. Paragangliomas are unusual neoplasms of the female genital tract, with few individual reported cases arising from the uterus, ovary, vagina, and cervix [2, 3]. Paraganglioma of the BL is exceedingly rare entity [4]. Most BL tumors present clinically with vague symptoms and are often detected either during a routine gynecological examination or on abdominal exploration because of the presence of a pelvic mass and vague lower abdominal discomfort or pain [5].

Here we present an unusual case of broad ligament paraganglioma, presenting as a cystic mass, in a 50-year-old postmenopausal female. A high degree of suspicion along with detailed immunohistopathological work-up is needed for arriving at an accurate diagnosis.

## 2. Case Report

A 50-year-old multiparous, postmenopausal female presented to gynecology outpatient department with burning micturition since one month. Physical examination revealed a blood pressure of 140/96 mmHg and a pulse rate of 76/minute. Vaginal examination revealed 8 cm × 8 cm cystic mass in the anterior fornix. Urine examination showed presence of pus cells. Other systemic examination and routine laboratory findings were noncontributory.

Pelvic ultrasonography (USG) showed well-defined large thick walled cystic mass measuring 7.6 cm × 6.8 cm in the left adnexa. Few incomplete septa were seen within it. Left ovary was not seen separately. Right ovary appeared to be normal. No free fluid was seen in the abdomen and pelvis. USG findings suggested the possibility of ovarian neoplastic mass. She was taken for exploratory laparotomy. Uterus and both ovaries were atrophic. Cystic mass measuring 8 cm × 6 cm was seen in the left broad ligament. Cyst was excised and sent for frozen section which showed cyst wall lined by sheets of tumor cells having eccentric vesicular nucleus and moderate amount of eosinophilic granular cytoplasm. Frozen section report of sex cord-stromal tumor was given. Complete excision of the lesion was advised for histopathological confirmation. Omental biopsy and peritoneal fluid were also sampled.

**Figure 1:**
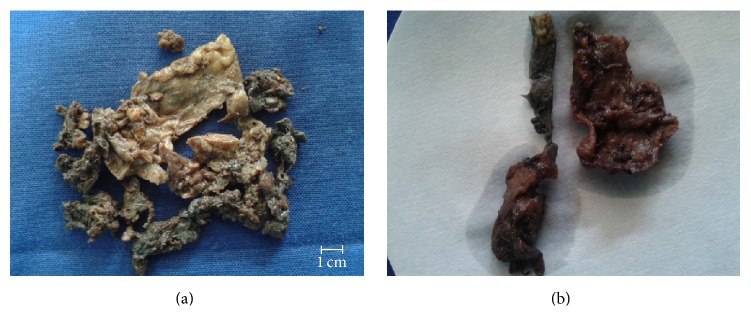
(a) Gross photograph of broad ligament paraganglioma. (b) Gross photograph of paraganglioma with dichromate reaction.

Gross examination showed presence of multiple flattened bits, all aggregating to 10 cc (Figure 1(a)). The external surface showed congested areas. The inner surface showed yellowish and hemorrhagic areas. The tumor areas showed positive dichromate reaction (done retrospectively) (Figure 1(b)).

**Figure 2:**
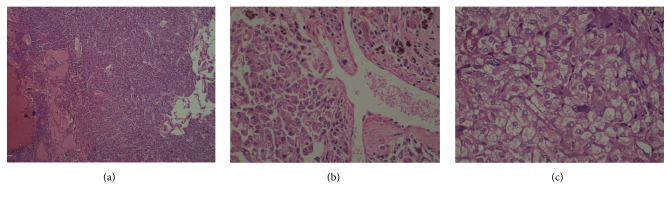
(a) Photomicrograph of broad ligament paraganglioma showing diffuse sheets of tumor cells having vesicular nuclei and moderate amount of eosinophilic granular cytoplasm were seen (H and E, ×100), (b) intracellular golden brown pigment (H and E, ×400), and (c) diffuse sheets of tumor cells having clear cytoplasm (H and E, ×400).

On microscopic examination, sheets of tumor cells having vesicular nuclei and moderate amount of eosinophilic granular cytoplasm (Figures 2(a) and 2(b)) and at places clear cytoplasm were seen (Figure 2(c)). Areas of hyaline acellular material were noted. The tumor was vascular and showed dilated and congested blood vessels. There was presence of intracellular golden brown hemosiderin pigment (Figure 2(b)). There was absence of nuclear pleomorphism, mitosis, and necrosis. Based on histomorphologic appearance, differential diagnosis of steroid cell tumor and paraganglioma was considered and representative sections were subjected for immunohistochemical (IHC) studies.

**Figure 3:**
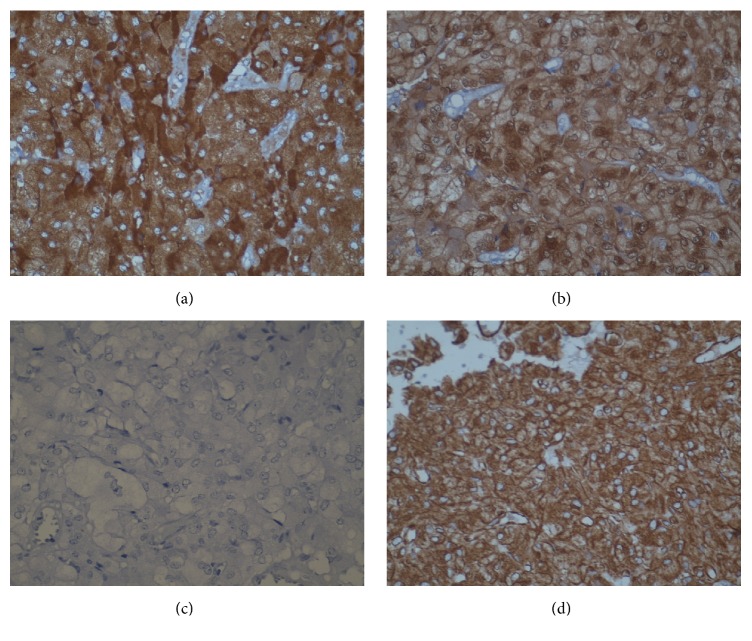
Immunohistochemical findings. (a) Tumor cells showing strong cytoplasmic immunoreactivity for chromogranin (×400). (b) Sustentacular cells showing strong nuclear and cytoplasmic immunoreactivity for S-100 (×400). (c) Tumor cells are nonimmunoreactive for Ki-67 (×400). (d) Tumor cells showing strong cytoplasmic immunoreactivity for vimentin (×400).

IHC was performed with the following panel of antibodies: Pancytokeratin (PanCK) (clone AE1/AE3, Dako), vimentin (clone V-9, Dako), EMA (clone E29, Dako), CD99 (clone 12E7, Dako), calretinin (5A5, LabIndia), inhibin (AMY82, LabIndia), S-100 (Leica), neuron-specific enolase (NSE) (Dako), chromogranin-A (clone 5H7, Leica), synaptophysin (clone 27G12, Leica), desmin (clone 33, Dako), smooth muscle actin (SMA) (clone 1A4, Novocastra), and S-100 (Leica). Hormonal status markers like estrogen receptor (ER) (clone 6F11, Novocastra) and progesterone receptor (PR) (clone PGR312, Novocastra) were also studied. Cell proliferation markers such as Ki-67 (clone MIB-1, Dako) and p53 (clone DO-7, Dako) were also included. The tumor cells showed strong cytoplasmic immunoreactivity for vimentin (Figure 3(d)), chromogranin (Figure 3(a)), synaptophysin, NSE, and S-100. The sustentacular cells showed cytoplasmic immunoreactivity for S-100 (Figure 3(b)). They were nonreactive for PanCK, EMA, CD99, calretinin, inhibin, desmin, SMA, ER, and PR. P53 was negative and Ki-67 labelling index (Ki-67 LI) was less than 1% (Figure 3(c)).

Pheochromocytoma of Adrenal Gland Scales Score (PASS) [6] was used for assessing prognosis in the present case. In view of diffuse growth pattern, PASS score of 2 was assigned in the present case. S-100 showed prominent immunoreactivity in sustentacular cells and tumor cells. Ki-67 labelling index was less than 1%. PASS score of 2, prominent S-100 immunoreactivity, and Ki-67 LI of less than 1% indicated likely benign behavior of the present tumor.

Based on histopathological and IHC studies, final diagnosis of paraganglioma of the BL was reached. The sections from omentum and peritoneal fluid cytology were unremarkable. Postoperatively, the patient is disease-free and on follow-up there is no evidence of recurrence for a period of six months.

## 3. Discussion

Although secondary involvement of the broad ligament by malignant tumors arising elsewhere in the abdomen is common, primary tumors in this location are rare. Paraganglioma of the BL is exceedingly rare. A Medline search of the English literature revealed only 4 cases of BL paraganglioma documented so far [4]. The criteria established by Gardener et al. for the tumor to be considered as arising from BL are that the tumor should be completely separable from and in no way connected to either uterus or ovary as seen in the present case [1].

Paragangliomas are derived from the extra-adrenal paraganglionic system, which is composed of cells from the neural crest which are associated with the autonomous nervous system. They are formed by chromaffin or nonchromaffin neural epithelioid cells, which are related to sympathetic or parasympathetic nerves. They are commonly found in abdomen, pelvis, head, and neck regions. Paragangliomas have rarely been reported in the female genital tract [2]. Possible theory of histogenesis of primary BL paraganglioma may include an origin from extra-adrenal paraganglia in the region of the BL.

Paragangliomas have the potential to present as a mass with paroxysmal symptoms such as palpitations, pallor, tremor, headache, and diaphoresis as well as hypertension due to their catecholamine secreting properties, observed in only 25% of the paragangliomas [7]. The current case presented as large cystic abdominal mass causing burning micturition. Thus, clinical manifestations of extra-adrenal pheochromocytomas are variable and nonspecific. The diagnosis therefore is difficult and depends on a high index of suspicion.

USG, computerized tomography (CT), and magnetic resonance imaging (MRI) are noninvasive and sensitive techniques in assessing BL tumors. Extra-adrenal paragangliomas have nearly identical imaging features, including a homogeneous or heterogeneous hyperenhancing soft tissue mass on CT and multiple areas of signal void interspersed with hyperintense foci within tumor mass on magnetic resonance imaging (MRI). An additional advantage of CT and MRI is their ability to evaluate sites of extra-adrenal pheochromocytomas [8]. It must be noted that the tumor in the present case showed extensive cystic degeneration converting it into a large cystic mass. Such presentation is not mentioned in the literature so far.

Although radiological modalities help in evaluating broad ligament tumors, immunohistopathology is mandatory in finally clinching the diagnosis and predicting its malignant potential. Careful assessment of routine histology may raise the suspicion of paraganglioma and prompt immunohistochemical evaluation leads to confirmation. Paragangliomas darken after exposure to potassium dichromate due to chromaffin reaction as seen in our case when done retrospectively [9]. Although histologically similar to the adrenal pheochromocytoma, the typical rounded “ball of cells” alveolar pattern (zellballen) may be less pronounced in the pelvic and extra-adrenal paragangliomas as seen in the present case [2]. Histopathological differential diagnoses of steroid cell tumor [10], luteinized granulosa cell tumor [11], and granular cell tumor [12] were considered in the present case. Steroid cell tumor shows cells with vacuolated cytoplasm. The tumor lacks stroma and shows inhibin and calretinin immunoreactivity but is negative for neuroendocrine markers. Luteinized granulosa cell tumor shows varying presence of nuclear grooves with vimentin, inhibin, calretinin, CD99, and SMA positivity with variable immunoreactivity for desmin and PanCK and negativity with EMA. Granular cell tumor shows S-100 positivity. Extra-adrenal pheochromocytoma shows NSE, chromogranin, and synaptophysin immunoreactivity. Sustentacular cells show prominent S-100 immunoreactivity [13]. There have been inhibin and calretinin positive paragangliomas reported so far. Positivity with these markers could constitute a diagnostic pitfall. However, neuroendocrine markers positivity helps in the confirmation of the diagnosis.

The following clinicopathological parameters may be taken into account for risk assessment of behavior of paragangliomas: location, size, PASS score [6], S-100 immunoreactivity [14], and Ki-67 labelling index [15]. PASS score includes histopathological parameters such as nuclear hyperchromasia and pleomorphism, capsular and vascular invasion, extension into adipose tissue, atypical mitotic figures, tumor cell spindling, cellular monotony, high cellularity, necrosis, and pattern of growth [8]. In the present case, PASS score of 2 was assigned in view of diffuse growth pattern. S-100 showed prominent immunoreactivity in sustentacular cells and tumor cells. Ki-67 labelling index was less than 1%. Taking into consideration all the above features, the present case was expected to behave in benign fashion.

Surgery for removal of the lesion remains the primary modality of treatment of paragangliomas. Due to the possibility of neoplasm recurrence and metastasis especially after incomplete surgical excision, patients need periodic checks and long-term follow-up [7].
